# Host Range and Genetic Diversity of Arenaviruses in Rodents, United Kingdom

**DOI:** 10.3201/eid1409.080209

**Published:** 2008-09

**Authors:** Kim R. Blasdell, Stuart D. Becker, Jane Hurst, Mike Begon, Malcolm Bennett

**Affiliations:** University of Liverpool, Liverpool, UK

**Keywords:** LCMV, rodents, disease vectors, serology, prevalence, variation genetics, dispatch

## Abstract

During a study to extend our knowledge of the host range and genetic diversity of arenaviruses in Great Britain, 66 of 1,147 rodent blood samples tested for antibody, and 127 of 482 tested by PCR, were found positive. All sequences most closely resembled those of previously identified lymphocytic choriomeningitis virus.

Viruses in the family *Arenaviridae* are separated into 2 distinct serocomplexes, the New World serocomplex and the Old World serocomplex (*1*). Several arenavirus species are known to cause human disease, including lymphocytic choriomeningitis virus (LCMV), which causes influenza-like clinical signs, occasionally with neurologic complications. Infection may be asymptomatic in up to one third of patients (*2*), and serious complications often occur in intrauterine infection (*3*). Less severe cases of adult human infection are likely underreported and often misdiagnosed (*4*).

LCMV is found worldwide, probably because of its association with its natural Old World host, the house mouse, *Mus musculus* (*5*). Although antibodies have also been detected in other rodent species (*6*,*7*), arenaviruses are known to be serologically cross-reactive. Few isolates of LCMV have been obtained from wild rodents so little is known about its genetic diversity. Recent studies on American arenaviruses found that diverse arenaviruses co-evolved with their rodent hosts (*8*), a finding that suggests that a more thorough study of European rodents might also identify novel arenaviruses. The purpose of this study was therefore to extend our knowledge of LCMV and LCMV-like arenaviruses in rodents in Great Britain.

## The Study

In total 1,147 blood samples were collected from rodents: 1,060 were live-trapped, wild animals from <20 sites (Table 1), and 87 blood samples were collected from a captive colony of wild house mice (*9*) and tested serologically. All animal research was conducted under license, according to UK regulations.

**Table 1:** Rodent species, numbers tested, seroprevalence, and viral RNA prevalence to LCMV at each UK and Republic of Ireland site*

Site code and year	Geographic location	Species tested for antibody	Species tested for viral RNA
PHF 2004	Cheshire	MMu (0/4), RN (0/2); SP = 0.0	
PHF 2005	Cheshire	***MMu (8/26)***; SP = 30.8	
BHF 2004	Cheshire	MMu (4/10); SP = 40.0	***MMu (2/9),*** RN (0/2) – 2 sequences
BHF 2005	Cheshire	***MMu (2/2)***, RN (0/2); SP = 50.0	
BGF	Cheshire	MMu (0/7), RN (0/2); SP = 0.0	
CLF 2004	Cheshire	MMu (0/6), RN (0/2); SP = 0.0	
CLF 2005	Cheshire	MMu (0/12), RN (0/4); SP = 0.0	
MF 2002	Cheshire	AS (0/10), MG (0/9), MA (0/2), MMu (0/30); SP = 0.0	***AS (1/10)***, MG (0/1), MA (0/1), ***MMu (2/4)*** – 3 sequences
MF 2004	Cheshire	***AS (4/10)***, MG (0/1), MA (0/1), ***MMu (2/4)***; SP = 37.5	
CZ 2002	Cheshire	AS (0/4), MG (0/4), ***CL (4/61)***, MMi (0/22), RN (0/2); SP = 4.3	***AS (1/1)***, CL (0/4), MMi (0/1), MMu (0/1) – 0 sequences
CZ spring 2003	Cheshire	AS (0/9), MG (0/3), MA (0/4), ***MMi (1/3)***, SP = 5.3	
CZ autumn 2003	Cheshire	*AS (1/18)*, MG (0/19), MA (0/4), MMu (0/1), SP = 2.9	
CZ 2004	Cheshire	***MMu (1/19)***, RN (1/12), SP= 3.2	
DF	Cheshire	***MMu (1/69)***, SP = 1.4	
MW	Cheshire	MG (0/105), AS (0/45), SP = 0.0	
RH	Cheshire	MG (0/19), AS (0/49), SP = 0.0	
LVFS	Cheshire	RN (0/2, SC (0/4), SP = 0.0	
FA	Merseyside	MA (0/2), AS (0/24), SP = 0.0	
KF	Northumberland	***MA (2/104)***, SP = 1.9	
LI	North Devon	RN (0/40), SP = 0.0	
IOW	Isle of Wight	***SV (1/18)***, SP = 5.6	
TF	Thetford	***SV (1/21)***, SP = 4.8	
CF	Cumbria	SC (0/10), SV, (0/4), SP = 0.0	
NI	Northern Ireland	***AS (1/149)***, SP = 0.7	
CA	Republic of Ireland	MG (0/15), SP = 0.0	
CC	Republic of Ireland	AS (0/7), SP = 0.0	
TW	Republic of Ireland	AS (0/10), SP = 0.0	
Other	Various locations	***RN (1/6)***, SC (0/1), ***SV (2/26)***, SP = NA	AS (0/1), MA (0/2), MMu (0/31), RN (0/5), ***SV (1/4)*** – 0 sequences
Captive colony	Captive colony, Cheshire	***MMu (30/87)***, SP = 34.5	***MMu* (122/403)** – 92 sequences

*LCMV, lymphocytic choriomeningitis virus; SP, site prevalence (%). Species key: MMu, *Mus musculus* (house mouse); RN, *Rattus norvegicus* (brown or Norway rat); AS, *Apodemus sylvaticus* (wood mouse); MG, *Myodes glareolus* (bank vole); MA, *Microtus agrestis* (field vole); MMi, *Micromys minutis* (harvest mouse); CL, *Cynomys ludovicianus* (black-tailed prairie dog); SV, *Sciurus vulgaris* (red squirrel); SC, *Sciurus carolensis* (gray squirrel); NA, not available. Species containing positive animals are in ***boldface italics,*** with number positive and number tested in parentheses.

Serum samples were separated by centrifugation (10,000 rpm, 10 min) and tested for LCMV antibody by using the manufacturer’s protocol for commercial indirect fluorescent antibody assay slides (Charles River Laboratories, Wilmington, MA, USA). A 1:40 dilution of anti-rat or anti-mouse immunoglobulin G fluorescein isothiocyanate (Sigma-Aldrich, Gillingham, UK) or a combination of both were used as secondary antibody. Ninety-three serum samples (from the original serum samples tested for antibody) that were either antibody positive or from sites with high seroprevalence were tested for arenavirus RNA by PCR. Another 379 blood samples from the captive colony of house mice, which had not been previously tested for antibody, were also tested. The PCR targeted a fragment of the glycoprotein precursor gene (GPC) (*10*). A selection of samples found negative by the GPC PCR were subsequently retested by PCR targeted at a fragment of the nucleoprotein (N) gene (*8*), by using primers to sequences common to the Old World arenaviruses. Total RNA was extracted by using QIAamp viral RNA mini-kit (QIAGEN, Crawley, UK), converted to cDNA, and amplified by using a single-step kit (Superscript III one-step RT-PCR with Platinum Taq polymerase system; Invitrogen, Paisley, UK) in conjunction with oligonucleotides arena1^+^ and LCMV322^–^ (*10*) or 1010C and either OW1696R or NW1696R (*8*). Products were separated and visualized by agarose gel electrophoresis, and amplicons were purified with the QIAquick PCR purification kit (QIAGEN). Bidirectional sequencing was performed off-site (MWG Biotech AG, Ebersberg, Germany). The 97-nt sequences generated here were deposited with GenBank (accession nos. DQ275199–DQ275295).

The software package MEGA version 4.0 (*11*) was used to construct an alignment of a 283-nt fragment of the GPC gene nucleotide sequences and predicted amino acid sequences, and for phylogenetic analysis with the neighbor-joining method (p distance model), with bootstrap support based on 1,000 pseudoreplicates. Other GenBank sequences included for comparison are listed in Table 2. Pairwise genetic distances were calculated by using the p distance model; percentage sequence identities were calculated by subtracting the genetic distances from 1.0 and multiplying by 100.

**Table 2:** Percentage nucleotide identities between the study sample sequences (all and from the captive colony only) and previously isolated LCMV and Lassa virus sequences*

Sequence	All study sequences, %	Captive colony sequences only, %
All study sequences	93.6–100	NS
Captive colony sequences only	NS	97.4–100
LCMV CIPV76001 Pasteur (AF095783; France)	78.7–80.5	78.7–80.5
LCMV CIP97001 (AF079517; France)	79.4– 83.1	80.9–83.1
LCMV Marseille (DQ286931; France)	82.8– 83.9	82.8–83.5
LCMV CH5871 (AF325215; Germany)	81.6–83.1	81.6–83.1
LCMV CH5692 (AF325214; Germany)	81.3–82.8	81.3–82.8
LCMV MX (EU195888; Slovakia)	78.7–80.5	78.7–80.5
LCMV Armstrong (M20869; USA)	82.8– 85.8	83.9–85.8
LCMV WE (M22138)	82.0–84.3	82.0–84.3
Lassa LP (AF181853)	58.1–59.9	58.8–59.9

*LCMV, lymphocytic choriomeningitis virus; NS, not shown.

Overall, 66 of 1,147 serum samples and 7 of 9 rodent species had antibodies to arenaviruses. *Sciurus vulgaris* had the highest prevalence, 26%, although only 15 squirrels were tested. *M. musculus* had the second highest prevalence, 17.5%. Antibodies were also detected in *Apodemus sylvaticus*, *Microtus agrestis*, *Micromys minutis*, captive-housed *Cynomys ludovicianus*, and *Rattus norvegicus*. Seroprevalence varied between species (1.4%–26%) and between sites (0%–50%) (Table 1).

GPC PCR amplicons were obtained from 127 of 472 tested samples, and sequences were determined for 97 samples (Table 1). All positive samples were from *Mus musculus* except 1 from *A. sylvaticus*. Twenty samples negative in the GPC PCR, but seropositive or from high prevalence sites, were tested by N gene PCR, and 2 were weakly positive: 1 *S. vulgaris* and 1 *A. sylvaticus*. In neither case, however, could a sequence be obtained from the amplicon.

Nucleotide and amino acid GPC sequence identities for all the samples in this study ranged from 93.6%–100% (Table 2), and 94.5%–100%, respectively (data not shown). When compared with other arenaviruses, the nucleotide sequences exhibited 78.7%–85.8% identity with LCMV reference sequences and only 58.1%–59.9% identity with Lassa virus (Appendix Figure).

Although antibodies to arenaviruses have been reported in a range of European rodent species, our study provided evidence of arenaviruses infecting red squirrels (*S. vulgaris*) and European harvest mice (*M. minutis*). Antibodies to arenaviruses have been reported in introduced *S. carolensis* in Great Britain (*12*) but were not detected in this study. We also reported antibodies to arenaviruses in black-tailed prairie dogs (*Cynomys ludovicianus*): those tested in this study were part of a colony in a zoo, however, and had contact with wild mice, some of which were seropositive. As found in previous studies, *Mus musculus* was more likely to be infected with LCMV than other rodent species.

The nucleotide sequences of most PCR amplicons clearly identified LCMV as the most frequent cause of the antibody detected. However, the detection of arenaviral RNA in 2 animals by the N gene PCR, but not by the LCMV-specific GPC PCR, may suggest the presence of another species of arenavirus. Further studies are needed to determine if other arenaviruses species are present in European rodent populations (*8*).

Genetic heterogeneity was present within and between sites (Figure), as seen in previous studies of arenaviruses (*13*,*14*). Sequences from animals in the captive colony and a nearby farm (MF) clustered and were different from those from a more distant farm (BHF). Furthermore, all of the British sequences clearly clustered separately from the reference strain sequences (from the United States, France, Germany, or Slovakia). These findings suggest spatial heterogeneity in sequence may be reflected in host range and pathogenicity. Sequencing might be useful in tracing sources of future human outbreaks.

**Figure:**
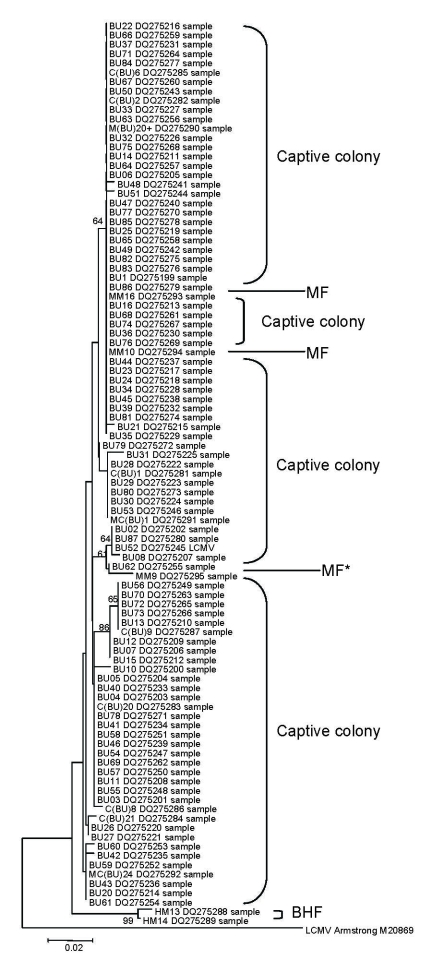
Unrooted neighbor-joining tree using the p-distance model (1,000 replicates) for a section of the glycoprotein precursor gene gene, showing bootstrap values of >60 for all sequences identified in this study (283 bp) and indicating site of origin. Captive colony, MF 2004, and BHF 2005 as in Table 1. MF* is from *Apodemus sylvaticus*, and all other sequences are from *Mus musculus*. Scale bar indicates number of substitutions per site.

## Conclusions

This study has increased the list of European (and North American) rodents that may be infected with LCMV and that might therefore pose a risk to humans. The genetic variation observed and potential variations in pathogenicity may indicate that some wildlife populations pose more of a public health risk than others. Further studies are needed to assess which mutations cause increased pathogenicity and to establish whether or not LCMV represents the only arenavirus present in European rodent populations.

## Supplementary Material

Appendix Figure Unrooted neighbor-joining radial tree that used the p-distance model (1,000 replicates) for a section of the glycoprotein precursor gene gene, rooted to Lassa virus strain LP. A total of 24 representative sequenced amplicons from wild rodents (283 bp) are shown, with comparisons to previously published lymphocytic choriomeningitis virus (LCMV) sequences and Lassa virus strain LP (GenBank). Scale bar indicates a distance of 0.05 substitutions per site.
