# Supplementary figures and images for: Host Range and Genetic Diversity of Arenaviruses in Rodents, United Kingdom
Source: Emerg Infect Dis. 2008 Sep;14(9):1455–8. doi: 10.3201/eid1409.080209 (PMC2603089; doi:10.3201/eid1409.080209)

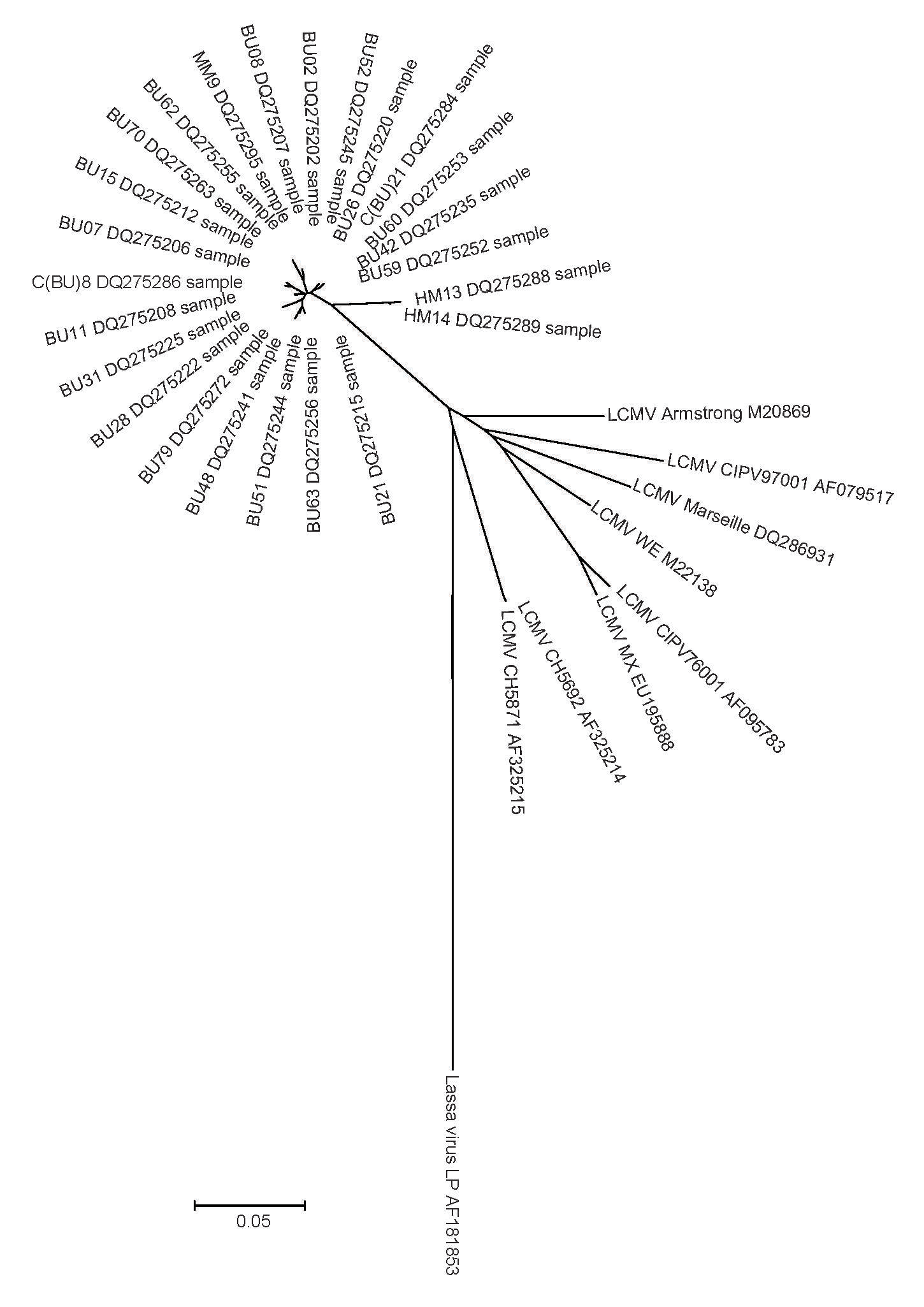

Supplement: Appendix Figure — Unrooted neighbor-joining radial tree that used the p-distance model (1,000 replicates) for a section of the glycoprotein precursor gene gene, rooted to Lassa virus strain LP. A total of 24 representative sequenced amplicons from wild rodents (283 bp) are shown, with comparisons to previously published lymphocytic choriomeningitis virus (LCMV) sequences and Lassa virus strain LP (GenBank). Scale bar indicates a distance of 0.05 substitutions per site. [file 08-0209_app-s1.gif]
